# Effect of Cholesterol on C99 Dimerization: Revealed by Molecular Dynamics Simulations

**DOI:** 10.3389/fmolb.2022.872385

**Published:** 2022-07-19

**Authors:** Cheng-Dong Li, Muhammad Junaid, Xiaoqi Shan, Yanjing Wang, Xiangeng Wang, Abbas Khan, Dong-Qing Wei

**Affiliations:** ^1^ State Key Laboratory of Microbial Metabolism, Joint Laboratory of International Cooperation in Metabolic and Developmental Sciences, Ministry of Education, Department of Bioinformatics and Biological Statistics, School of Life Sciences and Biotechnology, Shanghai Jiao Tong University, Shanghai, China; ^2^ Peng Cheng Laboratory, Shenzhen, China

**Keywords:** cholesterol, C99, dimerization, molecular dynamics simulations, *Alzheimer’s disease*

## Abstract

C99 is the immediate precursor for amyloid beta (Aβ) and therefore is a central intermediate in the pathway that is believed to result in Alzheimer’s disease (AD). It has been suggested that cholesterol is associated with C99, but the dynamic details of how cholesterol affects C99 assembly and the Aβ formation remain unclear. To investigate this question, we employed coarse-grained and all-atom molecular dynamics simulations to study the effect of cholesterol and membrane composition on C99 dimerization. We found that although the existence of cholesterol delays C99 dimerization, there is no direct competition between C99 dimerization and cholesterol association. In contrast, the existence of cholesterol makes the C99 dimer more stable, which presents a cholesterol binding C99 dimer model. Cholesterol and membrane composition change the dimerization rate and conformation distribution of C99, which will subsequently influence the production of Aβ. Our results provide insights into the potential influence of the physiological environment on the C99 dimerization, which will help us understand Aβ formation and AD’s etiology.

## Introduction

The aggregation of amyloid beta (Aβ) in the brain is the main cause of Alzheimer’s disease (AD). Aβ accumulation leads to a series of pathological changes, including neuronal death and amyloid plaque, which are pathologically characterized within the gray matter of an AD patient’s brain (Seubert et al., 1992; Iwatsubo et al., 1994; Kirkitadze et al., 2002; Walsh et al., 2002).

Aβ is cleaved by *γ-*secretase from the C99 domain of the amyloid precursor protein (C99), which is generated upon the cleavage of amyloid precursor protein by *ß*-secretase. Although great efforts have been made on the biophysical chemistry of Aβ, there are few studies on C99. Among the handful of articles, controversy has already arisen over whether and how it binds cholesterol, whether and how it dimerizes, and how these events relate to each other and to the *γ*-cleavage to release Aβ. Recent studies have also shown that C99 but not Aβ is a key contributor to early intraneuronal lesions in the triple-transgenic mouse hippocampus (Lauritzen et al., 2012); C99 but not Aβ is associated with the selective death of vulnerable neurons in AD. (Pulina et al., 2019) These findings highlight the importance of C99 in the pathogenesis of AD. (Pulina et al., 2019)

Early evidence has shown that elevated levels of cholesterol (CHOL) promote the amyloidogenic pathway, increasing Aβ production and inhibiting the competing nonamyloidogenic cleavage pathway (Bodovitz and Klein, 1996; Simons et al., 1998; Fassbender et al., 2001; Kojro et al., 2001; Refolo et al., 2001; Runz et al., 2002; Wahrle et al., 2002; Grimm et al., 2008; Guardia-Laguarta et al., 2009; Fonseca et al., 2010). In addition, studies found that 5–20 mol% cholesterol 2- to 4-fold enhances the rates of production of both Aβ40 and Aβ42 (Osenkowski et al., 2008). For the first time, Barrett et al. (2012) confirmed the association between C99 and cholesterol and proposed a cholesterol binding C99 model, where cholesterol forms a 1:1 binary complex with monomeric C99 at the repeat GxxxG motif. This finding hints at a significant functional effect of cholesterol on the C99 process and raises a hot debate on the relevance of C99 dimer in the *γ*-cleavage of C99.

Song et al. (2013) suggested that there is a competition between C99 homodimerization and the C99 cholesterol binding, since their binding interfaces both center on the G_700_xxxG_704_xxxG_708_ glycine–zipper motif and adjacent Gly_709_. They also suggested that cholesterol binding to C99 complex is more highly populated than C99 homodimers under most physiological conditions. However, increasing evidence suggests that C99 has multiple dimeric conformations, which are stabilized by different motifs (Munter et al., 2007; Gorman et al., 2008; Miyashita et al., 2009; Sato et al., 2009; Wang et al., 2011; Nadezhdin et al., 2012; Pester et al., 2013; Chen et al., 2014; Laura et al., 2014). Laura et al. found that C99 exists at least three configurations predominantly characterized by right-handed coiled coils including G-IN, G-SIDE, and GOUT (Laura et al., 2014). Our previous study found that C99 dimer exists six conformations regulated using the Helix switch (Li et al., 2019). In particular, instead of a 1:1 complex, we found that cholesterol molecules dynamically bind to the multiple sites of C99, whose binding affinity (Li et al., 2017) does not seem to be the ability to compete directly with C99 dimer (Li et al., 2019). Therefore, it raises the question of how dynamic cholesterol binding to multiple sites of C99 competes with C99 dimerization, which assembles into multiple dimeric conformations. In addition, studies have corroborated the view that C99 homodimerization is related to *γ*-cleavage (Scheuermann et al., 2001; Munter et al., 2007; Kienlen-Campard et al., 2008; Khalifa et al., 2010); disulfide-linked C99 homodimers have been shown to be cleaved to generate disulfide-bonded amyloid-β dimers (Scheuermann et al., 2001). These results challenge Song’s conclusion.

The contradiction above calls for further investigation of the effect of cholesterol on C99 dimerization, which is critical for our cognition of Aβ formation and the AD etiology since it will have a functional consequence on C99 dimerization and *γ*-cleavage. To answer these questions, a multiscale computational approach combining coarse-grained (CG) and all-atom (AA) simulations was employed to simulate two C99 monomers (PDB structure 2LLM) in POPC:POPG:CHOL = 3:1:1 and DPPC:CHOL = 4:1 bilayers with or without mol 20% cholesterol (to match the optimal concentration in Song’s experiments). (Song et al., 2013). CG simulations were performed for 3 μs using the MARTINI 2.2 force field (Marrink et al., 2007; Monticelli et al., 2008; Marrink et al., 2009; Jong et al., 2012) to assess the longtime dynamics of the protein and lipid conformation ensemble, whereas AA simulation was performed for 3 μs using CHARMM36 force field (Huang and MacKerell, 2013) to obtain more accurate forecasts for the association between C99 and cholesterol.

### Models and Methodology

The experimentally derived NMR structure 2LLM (Nadezhdin et al., 2012) (C99_15-55_) was employed as the initial structure of the C99 monomer. The protein sequence of C99_15-55_ is _696_GSNKGAIIGLMVGGVVIATVIVITLVMLKKK_726_, and G_700_-L_723_ is the transmembrane (TM) domain (TMD) of C99.

#### CG Model Simulations

The Martinize.py script was used to create protein topology information, and the CG simulations were performed under MARTINI v2.2 force field (Monticelli et al., 2008; Jong et al., 2012). The insane.py script was employed to build the POPC:POPG = 3:1 and DPPC bilayer systems (size = 8*8 nm^2^), with or without mol 20% cholesterol levels (where mol% cholesterol = 100[moles cholesterol/(moles lipids + moles cholesterol)]). Two spatially segregated monomers were then placed in the pre-equilibrated lipid systems. The CG bilayer system consisted of two C99 monomers; 240 DPPC or POPC:POPG (3:1) lipids, with or without 60 cholesterol molecules; 3863 water particles; and 6 Cl^−^ or 54 NA^+^ ions to neutralize the DPPC and POPC: POPG (3:1) lipid systems, respectively.

All CG systems experienced the following three steps: energy minimization, NVT and NPT equilibration, and the molecular dynamics balance. The energy of each system was repeatedly minimized followed by a 3 ns position-restrained simulation for better packing of the lipid molecules around the TM helices; 20 × 3 μs CG simulations were performed on each system in consideration of sample stability.

The temperature was set to 310 K using the V-rescale coupling method with a coupling constant of 1 ps. The pressure was set to 1 bar using a semiisotropic coupling for bilayer with the Berendsen algorithm. An integration time step of 20 fs was used in all simulations. Nonbonded interactions were truncated using shift functions (between 0.9 and 1.2 nm for Lennard–Jones interactions and between 0 and 1.2 nm for electrostatics) (Marrink et al., 2007; Jong et al., 2012).

### Restraint Coarse-Grained Simulations

To explore a possible cholesterol binding model of C99, restrained simulations of selected representative C99 dimeric conformation, which was obtained from our previous study, were performed in DPPC: CHOL = 4: 1 bilayer at 310 K, where the position of protein atoms was limited by the harmonic potential with the force constant of 1000 kJ mol^−1^nm^−2^ on each atom of the whole protein in the *X*, *Y*, and *Z* directions. During the simulations, the protein is almost stationary with tiny harmonic vibration of the side chains. The other physiological conditions and simulation parameters of the protein restrained simulations are the same as the above CG model simulations.

#### All-Atom Model Simulations

The AA model was constructed by the CHARMM-GUI Membrane Builder (Jo et al., 2009). The system is composed of C99 fragment (Gly_696_-Lys_726_), 240 DPPC molecules, 60 cholesterol molecules, 9756 water molecules, and 29 K^+^ and 35 Cl^−^ ions (0.15 M). Protein and lipids were presented using the CHARMM36 force field (Huang and MacKerell, 2013), and the TIP3P model (Jorgensen et al., 1983) was used for water. Two C99 monomers or representative C99 dimeric conformations (by converting CG to AA) were employed as the initial configuration. AA simulation system experienced the following three steps: energy minimization, NVT and NPT equilibration, and molecular dynamics simulation. The temperature was maintained at 310 K using the Nose–Hoover weak coupling algorithm (Hoover, 1985) with a coupling constant of 1 ps^−1^. The pressure was set to 1 bar using the Parrinello−Rahman barostat methodology (Parrinello and Rahman, 1998). The particle mesh Ewald algorithm (Darden et al., 1993) was applied in full electrostatics with a Fourier grid spacing of 0.12 nm. The van der Waals interactions were treated with a force-based switching function, with a switching range of 1.0–1.2 nm. All the covalent bonds that included hydrogen were restrained using the LINCS method (Hess et al., 2008). Periodic boundary conditions were employed in all three directions, and 2 fs integration time steps were used in AA simulations.

#### Binding Energy Calculation

g_mmpbsa (Baker et al., 2001; Kumari et al., 2014) was used to evaluate binding energies as well as to estimate the energy contribution of each residue to the binding energy. The binding energy is calculated based on the equation: ∆G_bind_ = ∆E_MM_ + ∆G_psolv_ + ∆G_npsolv_ − T∆S. ∆E_MM_ is the sum of van der Waals and electrostatic interactions. The entropy contribution (T∆S) is not calculated in the g_mmpbsa, and since the C99 is a transmembrane protein, we did not calculate the solvation energy. Therefore, this binding energy is the relative binding energy rather than the absolute binding energy (Vashisht et al., 2016). All energy components for the binding complex were averaged by 20 calculations from the production trajectory, where the complex is relatively stable.

The simulations were performed and analyzed using the GROMACS (v4.6.3) (Hess et al., 2008)^,^ (Van Der Spoel et al., 2005) Images were generated using VMD. (Humphrey et al., 1996). Detailed simulations are summarized in Supplementary Table S1. Note: The words “POPG” in legends refer to “POPC:POPG bilayer.”

## Results and Discussion

### Cholesterol Delays C99 Dimerization

**FIGURE 1:**
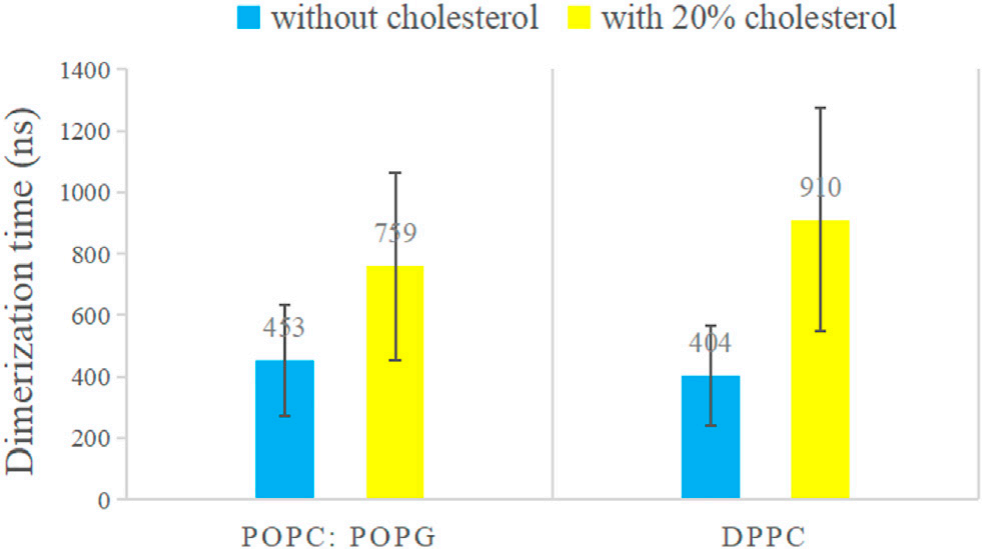
Average dimerization time of C99 dimer in POPC:POPG bilayer and DPPC bilayer with (orange) or without (blue) 20% cholesterol, from the average of 20 samples. The values are labeled in the corresponding areas. The dimerization time of C99 dimer was calculated based on the center of mass (COM) distance between the C99 monomers. When the COM distance between the two C99 TMDs (transmembrane domains) maintains a level below 1 nm continuously, we judge that the C99 monomers form a stable dimer.

All simulation replicas were observed to undergo a spontaneous conversion from two separated C99 monomers to a stable dimer that subsequently never disassociates. However, the speed of C99 dimerization differs with the change in membrane composition (Figure 1). The average dimerization time of C99 in 20% cholesterol (759 ns in POPG, 910 in DPPC) is significantly higher than that without cholesterol (453 ns in POPG, 404 ns in DPPC) (Figure 1). These results show that cholesterol delays the dimerization of C99.

Song et al., (Song et al., 2013) performed experiments around room temperature of 298 K in POPC:POPG bilayer with 20% cholesterol and observed that the presence of cholesterol results in an increase in the proportion of C99 monomers relative to C99 dimers. They therefore proposed that there is a competition between C99 homodimerization and C99 cholesterol binding, and the complex of cholesterol binding to C99 is more highly popular than C99 homodimers under most physiological conditions. However, CG simulations show that C99 monomers dimerize naturally in cholesterol environment in all replicas (Figure 1). In addition, we performed a 3 μs AA simulation of C99 monomers with 20% cholesterol, where the initial two separated C99 monomers are preset with one cholesterol molecule bound to its GxxxG motif (this structure is obtained from our previous work) to mimic the 1:1 complex of C99 cholesterol. During the simulation, the two cholesterol molecules left the GxxxG motif site successively, and C99 monomers form a dimer finally (Supplementary Figure S1). Computational results suggest that there is no direct competition between cholesterol association and C99 dimerization.

**FIGURE 2:**
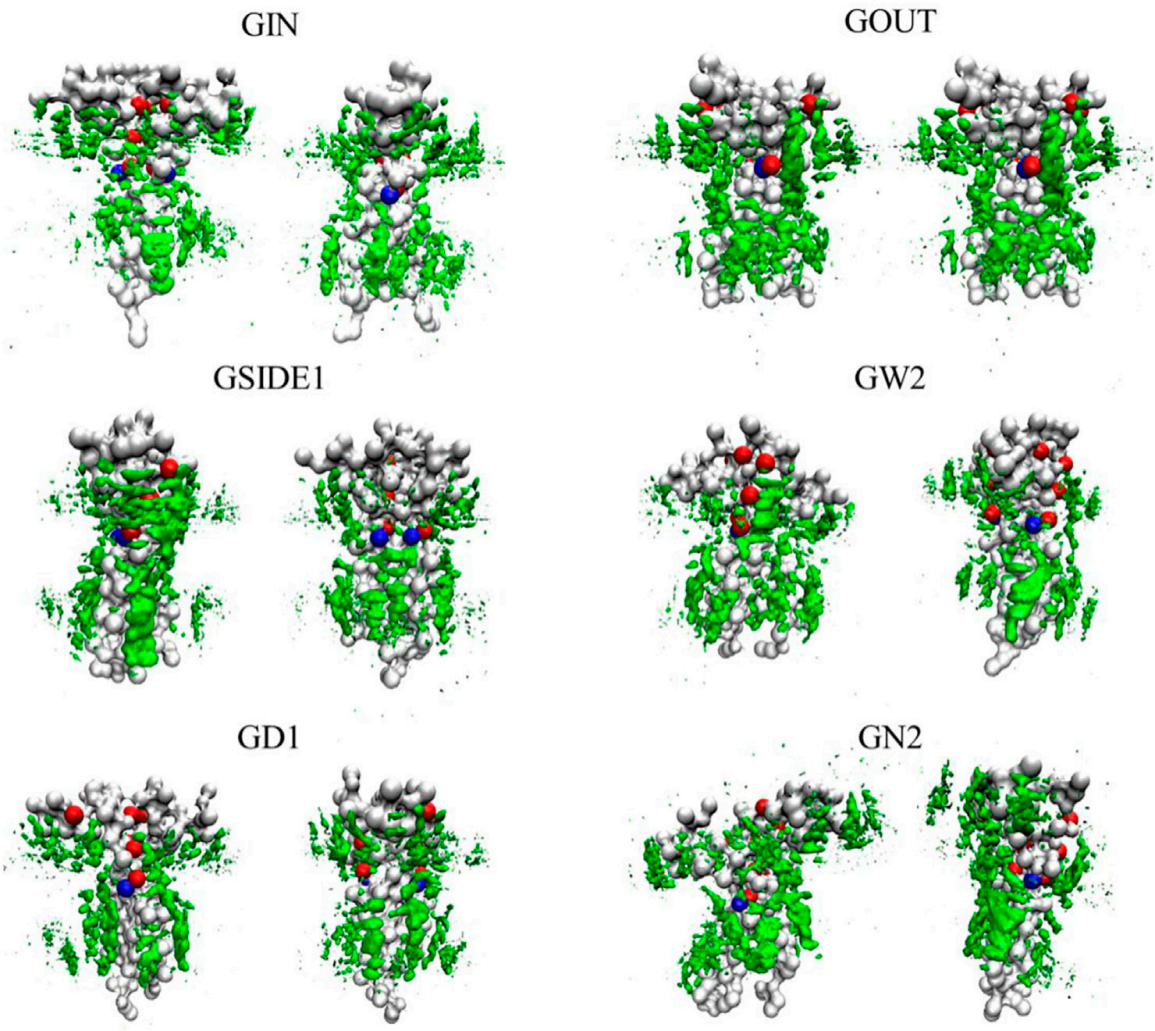
Cholesterol isosurfaces based on the restrain simulations of the representative C99 dimeric conformations, which were derived from our previous study (Li et al., 2019). Gly residues are shown in red beads; Ala residues are shown in blue beads; cholesterol isosurfaces are shown on green surface.

Song’s inference is based on the previous knowledge that 1) both C99 dimer binding interface and C99 cholesterol binding interface center on the GxxxG motif and that 2) cholesterol strongly binds to the GxxxG motif of C99, which is derived under experimental condition pH = 4.5. However, Straub et al. have suggested that the binding stability of cholesterol at the GxxxG motif critically depends on the protonation states of Glu693 and Asp694, which therefore is sensitive to pH. Under neutral pH conditions, the strong binding of cholesterol at the GxxxG motif will be deprived (Panahi et al., 2016). Our previous study further revealed that cholesterol molecules dynamically instead of strongly bind to the multiple sites of C99 (Li et al., 2017). In addition, recent studies have corroborated the view that C99 dimer exists multiple conformations regulated by different interfaces. This means that the C99 dimer binding interface and the C99 cholesterol binding interface do not necessarily overlap. Song’s experimental results can be interpreted by our computational observation that cholesterol delays C99 dimerization, which may be caused by 1) the cholesterol interaction with C99 or/and 2) the effect of cholesterol on the microenvironment of the membrane. A high concentration of cholesterol would reduce the fluidity of the membrane and increase the rigidity of the membrane, thus slowing down the molecular movement and C99 dimerization, leading to an increase in the proportion of C99 monomers relative to C99 dimers on the macro level. Since C99 assembles into a variety of dimer conformations and has multiple cholesterol dynamic binding sites, a dynamic multisite cholesterol binding model of C99 dimer is more reasonable, as shown in Figure 2.

### Cholesterol Binding Model of C99 Dimer

To explore the possible cholesterol association with C99 dimer, we carried out the restraint simulations of different C99 dimeric conformations respectively, where the C99 dimer was restrained while other molecules normally move. In the simulations, C99 dimer performed as a flower with multiple bees of cholesterol molecules dynamically circling around, binding to, and flying away from time to time. The flip-flop of cholesterol molecules over the upper and lower layers occurs occasionally. According to the density statistics of cholesterol, six different cholesterol isosurfaces were statistically drawn out based on the selected representative C99 dimeric conformations. As shown in Figure 2, there is dense cholesterol aggregation around each C99 dimeric conformation. These diverse cholesterol binding models verify the existence of multiple cholesterol binding sites of C99 and suggest coexistence rather than a competition relationship between C99 dimer and the cholesterol binding. As revealed in our previous studies that cholesterol molecules dynamically bind to the multiple sites of C99, and C99 dimer exists six conformations regulated using Helix switch. Therefore, once C99 forms a certain dimeric conformation, cholesterol would bind to else available sites of C99 dimer, forming a model of cholesterol surrounding C99 dimer (Figure 2). The association between C99 dimer and cholesterol will promote the partition of C99 dimer into raft-like membrane domains where C99 is more likely to encounter *ß*- and *γ*-secretase (β- and *γ*-secretase preferentially associate with the lipid raft) and less likely to come across α-secretase. In addition, such dynamic cholesterol binding allows cholesterol molecules to move away and will not be a steric hindrance during the processing of *γ*-cleavage of C99.

### Existence of Cholesterol Makes C99 Dimer More Stable

**FIGURE 3:**
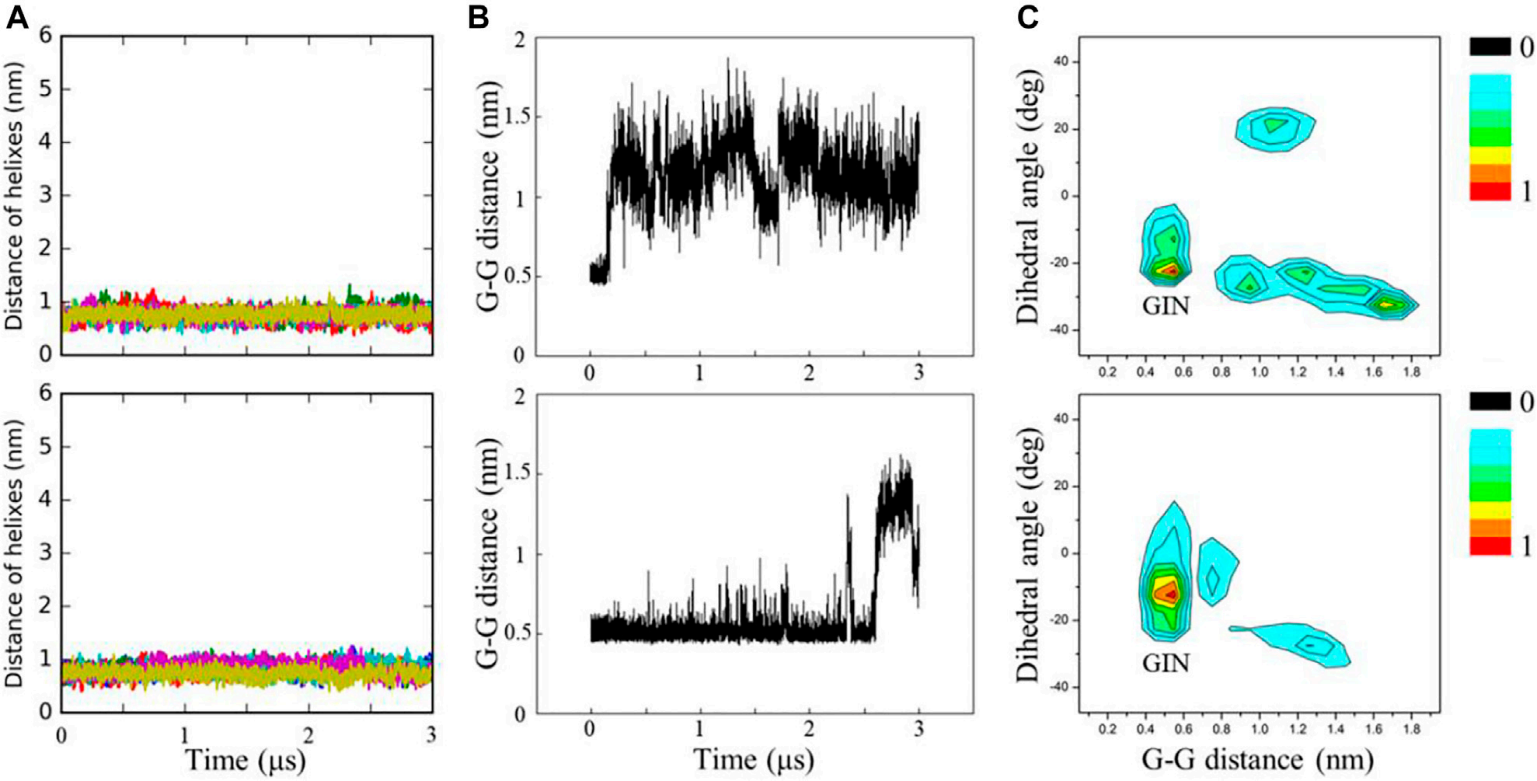
**(A)** COM distance between C99 monomers as a function of time. **(B)** COM distance between the two specified residues G19 and G19′ as a function of time. **(C)** Conformation distributions for C99 homodimer were projected onto the two parameters of Ω and G−G. Ω is the interhelix dihedral angle of C99 dimer; G−G is the distance between G19 and G19′. The data are from the simulations initiated with GIN conformation with (bottom) or without cholesterol (top). The colored scale on the right defines the relative population.

**FIGURE 4:**
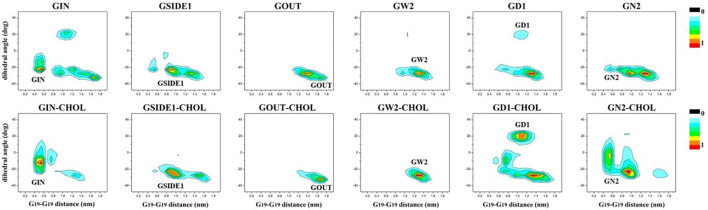
The conformation distributions for C99 homodimer from the simulations starting with six representative conformations in DPPC bilayer were projected onto the two parameters of Ω and G−G. The top panel is the distribution of C99 dimer when without cholesterol, whereas the bottom panel is the distribution when with 20% cholesterol. The representative structures are labeled in the corresponding regions.

To assess the effect of cholesterol on the stability of C99 dimer, we performed the 3 μs CG simulations starting from representative C99 dimeric conformations (GIN, GSIDE1, GOUT, GW2, GD1, and GN2), which were derived from our previous study (Li et al., 2019). It was observed in all replicas that C99 dimer never disassociates throughout simulations no matter with or without cholesterol (Figure 3A). This indicates the stability of C99 dimer in the above environments. Taking GIN as an example, we find GIN in cholesterol is more stable than that without cholesterol, and the presence of cholesterol postpones the time of GIN changing to other conformations (Figure 3B). When without cholesterol, GIN quickly shifts to other conformations in ∼0.2 μs, whereas in cholesterol environment, C99 dimer keeps GIN conformation until ∼2.5 μs. As a result, GIN conformation (G_19_−G_19_ ≈ 0.5, Ω ≈ −21°) is more highly distributed in cholesterol environment than that without cholesterol environment (Figure 3C). This phenomenon is consistently observed in the simulations starting with the representative dimeric conformations (GIN, GSIDE1, GOUT, GW2, GD1, and GN2). The population of the initial C99 dimeric conformation in cholesterol environment is denser than that in no cholesterol environment (Figure 4). Furthermore, we calculated the MM energy of GW2 from the AA simulations when with and without cholesterol. The energy analysis shows that GW2 has a more favorable binding energy in cholesterol than that without cholesterol (Supplementary Figure S2). These results suggest that the existence of cholesterol makes C99 dimer more stable and delays the mutual transformation of C99 dimeric conformations.

### C99 Dimeric Conformation Distribution is Affected by Membrane Composition

**FIGURE 5:**
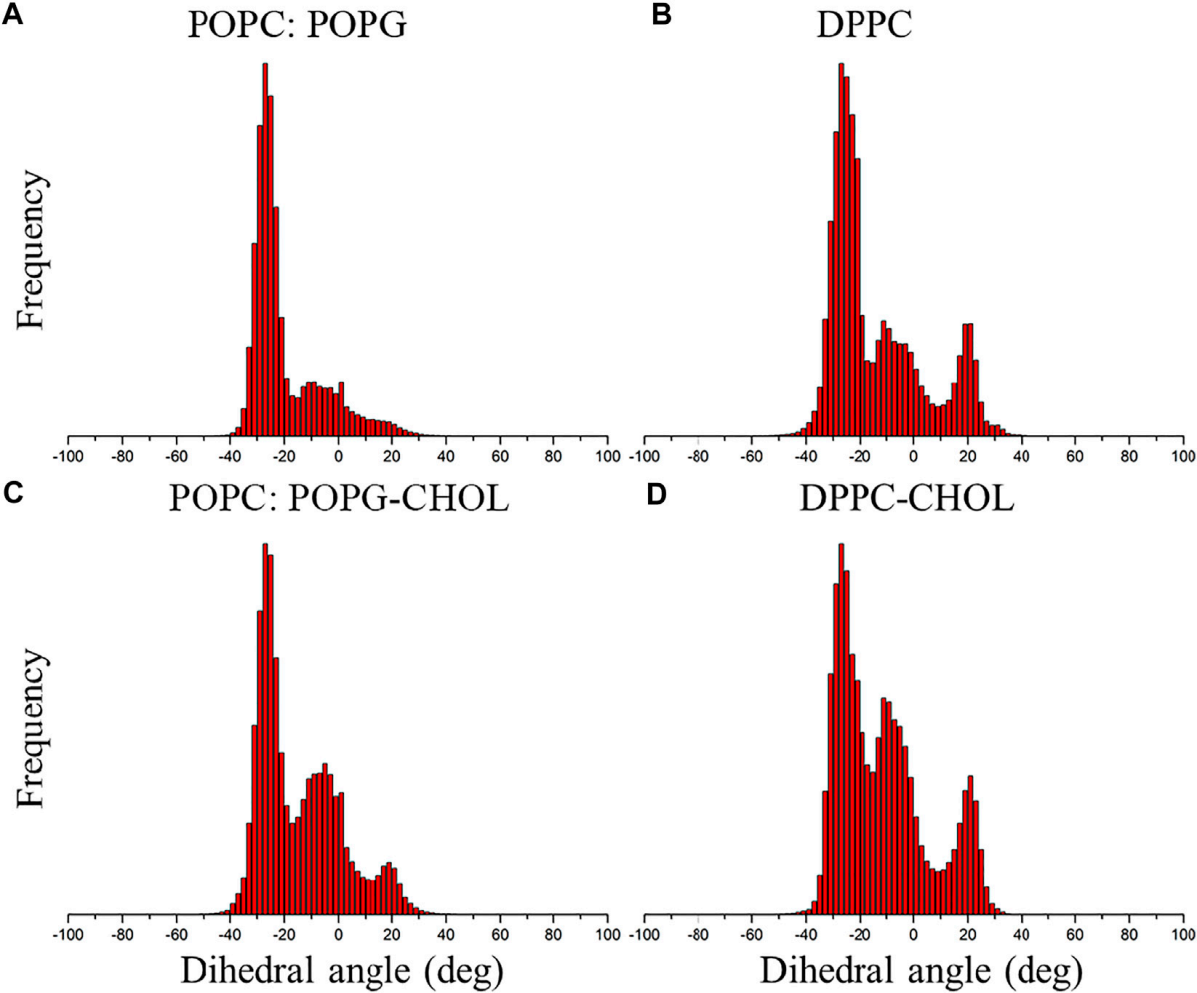
Normalization of the frequency of the dihedral angle of C99 dimer. **(A)** In POPC:POPG bilayer without cholesterol. **(B)** In DPPC bilayer without cholesterol. **(C)** In POPC:POPG bilayer with 20% cholesterol. **(D)** In DPPC bilayer with 20% cholesterol.

To compare the C99 dimer ensemble in different conditions, we calculated the dihedral angle of C99 dimer. The statistical results show that C99 dimer strongly favors a right-handed helical packing with a dihedral angle of ≈−25° to −30° (Figure 5), which matches the previous studies. In addition, there are two kinds of helical packings of ≈−5° and ≈20° in the structural ensemble. Statistical results show that the helical packings of ≈−5° and ≈20° are more popular in cholesterol than that in no cholesterol environment and are more popular in DPPC bilayer than that in POPC:POPG bilayer (Figure 5). These results indicate that membrane composition changes the distribution of C99 dimeric conformations.

**FIGURE 6:**
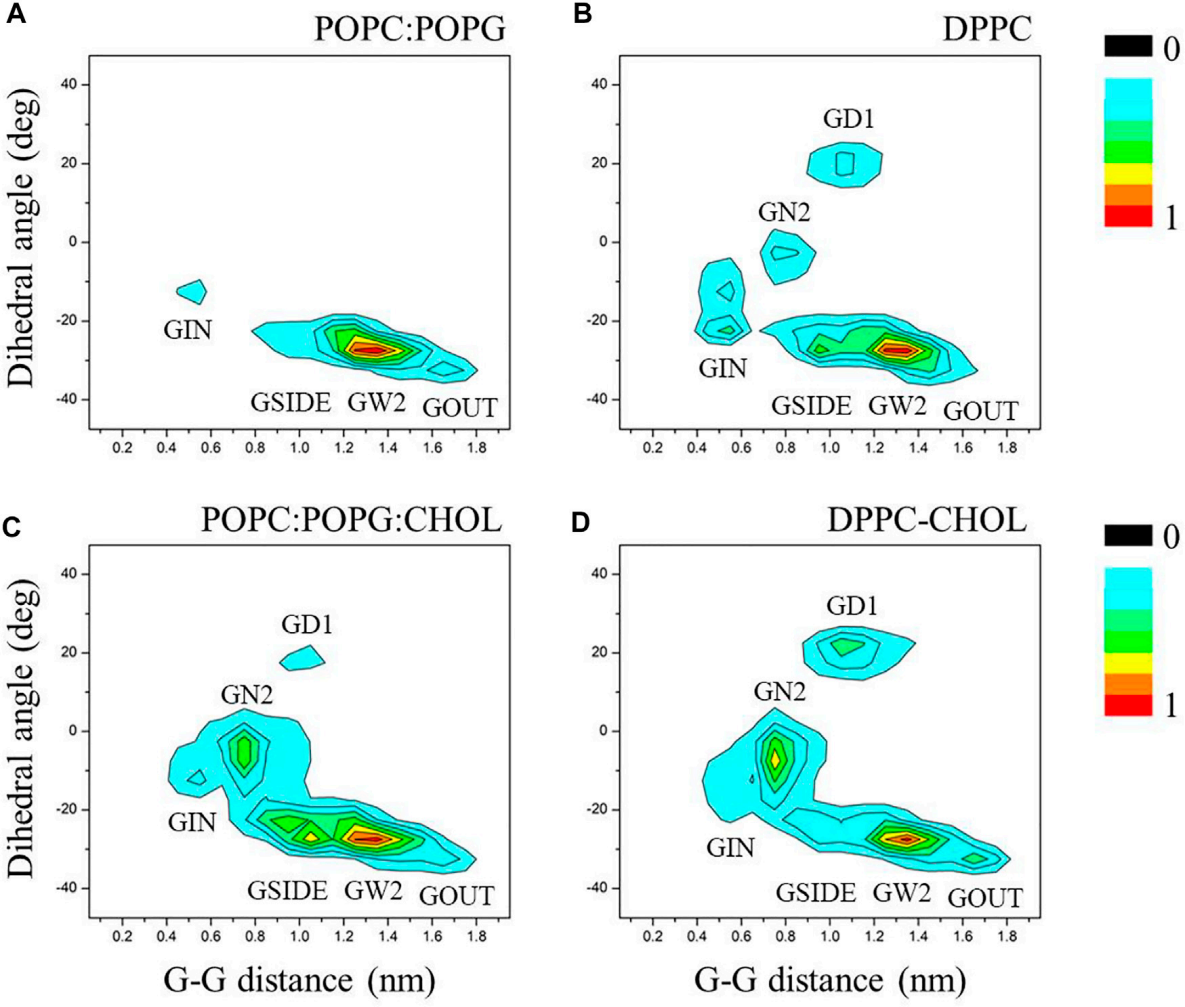
The distributions for C99 homodimer were projected onto the order parameters of Ω and G−G, using the data collected after C99 forms a dimer. **(A)** In POPC:POPG bilayer without cholesterol. **(B)** In DPPC bilayer without cholesterol. **(C)** In POPC:POPG bilayer with 20% cholesterol. **(D)** In DPPC bilayer with 20% cholesterol.

To further investigate the effect of membrane composition on the C99 dimerization, we characterize the distribution of C99 dimer by the two feature vectors. Statistical results show that GW2 is the predominant conformation both in POPC:POPG and DPPC bilayers whether with or without cholesterol (Figure 6). The membrane components show a selectivity toward C99 dimer. Compared with DPPC bilayer, there are no GD1 and GN2, and GIN takes a very low proportion in POPC:POPG bilayer, which presents a more compact energy landscape. This difference may be caused by the charged membrane composition. Studies have shown that proteins interact with the charged membrane (Lorenz et al., 2008; Brehmer et al., 2012; Yanez Arteta et al., 2014), our previous study has also shown that C99 monomer closely interacts with the charged POPG lipids (Li et al., 2019). TM helixes are sensitive to lipid properties such as the hydrophobic thickness and head group charge, and they adopt different packing to adapt to the lipids (Sapay et al., 2010), which therefore changes the C99 dimeric conformation distribution. On the other hand, the presence of cholesterol enriches the conformation assembly. As shown in Figures 3 and 4, cholesterol makes C99 dimer more stable and delays the mutual transformation of C99 dimeric conformations, which results in a more equitable distribution of each conformation and makes the energy landscapes between the two bilayers closer (Figure 6).

## Association Between C99 Dimerization and Amyloid-Beta

At least six types of Aβ peptides have been found so far, varying in length from 38 to 43 residues (Czirr et al., 2008). The longer Aβ (particularly the Aβ42 and Aβ43) are highly prone to oligomerization because of their stronger hydrophobic affinity. Soluble Aβ protein has been identified as the dark hand behind AD pathology (Bernstein et al., 2009; Bucciantini et al., 2002; Butterfield and Lashuel, 2010; Glabe, 2008; Klein et al., 2001). Recent studies have further identified its real form, the dimer of Aβ (Zott et al., 2019a). Aβ dimers undermine neuronal function by interfering with the reuptake of extracellular glutamate (Shankar et al., 2008; Zott et al., 2019b; Selkoe, 2019).

On the other hand, studies have proposed that C99 homodimerization is directly related to *γ-*cleavage and Aβ formation (Scheuermann et al., 2001; Munter et al., 2007; Kienlen-Campard et al., 2008; Khalifa et al., 2010). The disulfide-linked C99 homodimers have been shown to be cleaved to generate disulfide-bonded Aβ dimers (Scheuermann et al., 2001). This leads to the question of whether different C99 dimeric conformations produce different Aβ peptides or even Aβ dimers *via γ-*cleavage. In our previous research, we identified six conformation types of the C99 dimer and discussed that different C99 dimeric conformations with differential exposures ofγ-cleavage sites and insertion depths may modulate the γ-cleavage of C99, leading to different Aβ levels (cleavage of GIN may produce Aβ42 and cleavage of GOUT may produce Aβ40). In this study, we find that cholesterol and membrane composition influence the C99 dimerization rate and C99 dimeric conformation distribution. Cholesterol partitioning has been shown to occur with relevance to the affinity of cholesterol for different phospholipids (Halling et al., 2008). Therefore, the dynamic details of how cholesterol affects C99 assembly will be firstly determined by the membrane composition and how it subsequently influences the C99 dimerization and the *γ*-cleavage of C99, leading to a different production of Aβ.

Overall, our results present a detailed description of C99 dimerization in different physiological environments, which helps us understand AD’s etiology. Nevertheless, we recognize that our results are limited by the current simulation conditions (physiological environments, force field, and crystal structure), especially in the context that CG simulations are highly dependent on the definition of secondary structure, and only segmental crystal structures of C99 are available. Therefore, our simulations attempt to reveal but cannot fully describe the effect of cholesterol on C99 dimerization. These computational clues await further experimental examination.

## Conclusions

We investigated the effect of membrane composition on C99 dimerization, which influences C99 dimerization speed and the conformation distribution of C99 dimer, thus subsequently changing the level of Aβ or even Aβ dimer. Although the existence of cholesterol delays C99 dimerization, there is no direct competition between C99 dimerization and cholesterol association. By contrast, cholesterol makes C99 dimer more stable, presenting a cholesterol binding C99 dimer model. Membrane composition shows a selectivity toward C99 dimeric conformations. Compared with DPPC bilayer, a more compact energy landscape was observed in POPC:POPG bilayer. The presence of cholesterol enriches the conformation assembly, leading the energy landscapes between the two bilayers closer. Our results provide insights into the potential influence of the physiological environment on the C99 dimerization and new clues to understanding the mechanism of Aβ production and the pathogenesis of AD.

## Data Availability

The raw data supporting the conclusion of this article will be made available by the authors, without undue reservation.

## References

[B1] Baker N. A. Sept D. Joseph S. Holst M. J. McCammon J. A. (2001). Electrostatics of Nanosystems: Application to Microtubules and the Ribosome. Proc. Natl. Acad. Sci. U.S.A. 98, 10037–10041. 10.1073/pnas.181342398 11517324 PMC56910

[B2] Barrett P. J. Song Y. Van Horn W. D. Hustedt E. J. Schafer J. M. Hadziselimovic A. (2012). The Amyloid Precursor Protein Has a Flexible Transmembrane Domain and Binds Cholesterol. Science 336, 1168–1171. 10.1126/science.1219988 22654059 PMC3528355

[B3] Bernstein S. L. Dupuis N. F. Lazo N. D. Wyttenbach T. Condron M. M. Bitan G. (2009). Amyloid-β Protein Oligomerization and the Importance of Tetramers and Dodecamers in the Aetiology of Alzheimer's Disease. Nat. Chem. 1, 326–331. 10.1038/nchem.247 20703363 PMC2918915

[B4] Bodovitz S. Klein W. L. (1996). Cholesterol Modulates α-Secretase Cleavage of Amyloid Precursor Protein. J. Biol. Chem. 271, 4436–4440. 10.1074/jbc.271.8.4436 8626795

[B5] Brehmer T. Kerth A. Graubner W. Malesevic M. Hou B. Brüser T. (2012). Negatively Charged Phospholipids Trigger the Interaction of a Bacterial Tat Substrate Precursor Protein with Lipid Monolayers. Langmuir 28, 3534–3541. 10.1021/la204473t 22263701

[B6] Bucciantini M. Giannoni E. Chiti F. Baroni F. Formigli L. Zurdo J. (2002). Inherent Toxicity of Aggregates Implies a Common Mechanism for Protein Misfolding Diseases. Nature 416, 507–511. 10.1038/416507a 11932737

[B7] Butterfield M. Lashuel A. (2010). Amyloidogenic Protein–Membrane Interactions: Mechanistic Insight from Model Systems. Angew. Chem. 49, 5628–5654. 20623810 10.1002/anie.200906670

[B8] Chen W. Gamache E. Rosenman D. J. Xie J. Lopez M. M. Li Y.-M. (2014). Familial Alzheimer's Mutations within APPTM Increase Aβ42 Production by Enhancing Accessibility of ε-cleavage Site. Nat. Commun. 5, 3037. 10.1038/ncomms4037 24390130 PMC4082030

[B9] Czirr E. Cottrell B. A. Leuchtenberger S. Kukar T. Ladd T. B. Esselmann H. (2008). Independent Generation of Aβ42 and Aβ38 Peptide Species by γ-Secretase. J. Biol. Chem. 283, 17049–17054. 10.1074/jbc.m802912200 18426795

[B10] Darden T. York D. Pedersen L. (1993). Particle Mesh Ewald: AnN Log(N) Method for Ewald Sums in Large Systems. J. Chem. Phys. 98, 10089–10092. 10.1063/1.464397

[B11] Fassbender K. Simons M. Bergmann C. Stroick M. Lütjohann D. Keller P. (2001). Simvastatin Strongly Reduces Levels of Alzheimer's Disease β-amyloid Peptides Aβ42 and Aβ40 *In Vitro* and *In Vivo* . Proc. Natl. Acad. Sci. U.S.A. 98, 5856–5861. 10.1073/pnas.081620098 11296263 PMC33303

[B12] Fonseca A. C. R. G. Resende R. Oliveira C. R. Pereira C. M. F. (2010). Cholesterol and Statins in Alzheimer's Disease: Current Controversies. Exp. Neurol. 223, 282–293. 10.1016/j.expneurol.2009.09.013 19782682

[B13] Glabe C. G. (2008). Structural Classification of Toxic Amyloid Oligomers. J. Biol. Chem. 283, 29639–29643. 10.1074/jbc.r800016200 18723507 PMC2573087

[B14] Gorman P. M. Kim S. Guo M. Melnyk R. A. McLaurin J. Fraser P. E. (2008). Dimerization of the Transmembrane Domain of Amyloid Precursor Proteins and Familial Alzheimer's Disease Mutants. BMC Neurosci. 9, 17. 10.1186/1471-2202-9-17 18234110 PMC2266763

[B15] Grimm M. O. W. Grimm H. S. Tomic I. Beyreuther K. Hartmann T. Bergmann C. (2008). Independent Inhibition of Alzheimer Disease β- and γ-Secretase Cleavage by Lowered Cholesterol Levels. J. Biol. Chem. 283, 11302–11311. 10.1074/jbc.m801520200 18308724

[B16] Guardia-Laguarta C. Coma M. Pera M. Clarimón J. Sereno L. Agulló J. M. (2009). Mild Cholesterol Depletion Reduces Amyloid-β Production by Impairing APP Trafficking to the Cell Surface. J. Neurochem. 110, 220–230. 10.1111/j.1471-4159.2009.06126.x 19457132 PMC2741735

[B17] Halling K. K. Ramstedt B. Nyström J. H. Slotte J. P. Nyholm T. K. (2008). Cholesterol Interactions with Fluid-phase Phospholipids: Effect on the Lateral Organization of the Bilayer. Biophys. J. 95, 3861–3871. 10.1529/biophysj.108.133744 18641061 PMC2553118

[B18] Hess B. Kutzner C. van der Spoel D. Lindahl E. (2008). GROMACS 4: Algorithms for Highly Efficient, Load-Balanced, and Scalable Molecular Simulation. J. Chem. Theory Comput. 4, 435–447. 10.1021/ct700301q 26620784

[B19] Hoover W. G. (1985). Canonical Dynamics: Equilibrium Phase-Space Distributions. Phys. Rev. A 31, 1695–1697. 10.1103/physreva.31.1695 9895674

[B20] Huang J. MacKerell A. D. (2013). CHARMM36 All-Atom Additive Protein Force Field: Validation Based on Comparison to NMR Data. J. Comput. Chem. 34, 2135–2145. 10.1002/jcc.23354 23832629 PMC3800559

[B21] Humphrey W. Dalke A. Schulten K. (1996). VMD: Visual Molecular Dynamics. J. Mol. Graph. 14, 33–38. 10.1016/0263-7855(96)00018-5 8744570

[B22] Iwatsubo T. Odaka A. Suzuki N. Mizusawa H. Nukina N. Ihara Y. (1994). Visualization of Aβ42(43) and Aβ40 in Senile Plaques with End-specific Aβ Monoclonals: Evidence that an Initially Deposited Species Is Aβ42(43). Neuron 13 (43), 45–53. 10.1016/0896-6273(94)90458-8 8043280

[B23] Jo S. Lim J. B. Klauda J. B. Im W. (2009). CHARMM-GUI Membrane Builder for Mixed Bilayers and its Application to Yeast Membranes. Biophysical J. 97, 50–58. 10.1016/j.bpj.2009.04.013 PMC2711372 19580743

[B24] Jong D. D. Singh G. Bennett W. Arnarez C. Wassenaar T. A. Periole X. (2012). Improved Parameters for the Martini Coarse-Grained Protein Force Field. J. Chem. Theory Comput. 9. 10.1021/ct300646g 26589065

[B25] Jorgensen W. L. Chandrasekhar J. Madura J. D. Impey R. W. Klein M. L. (1983). Comparison of Simple Potential Functions for Simulating Liquid Water. J. Chem. Phys. 79, 926–935. 10.1063/1.445869

[B26] Khalifa N. B. Hees J. V. Tasiaux B. Huysseune S. Smith S. O. Constantinescu S. N. (2010). What Is the Role of Amyloid Precursor Protein Dimerization? Cell Adhesion Migr. 4, 268–272. 10.4161/cam.4.2.11476 PMC2900624 20400860

[B27] Kienlen-Campard P. Tasiaux B. Van Hees J. Li M. Huysseune S. Sato T. (2008). Amyloidogenic Processing but Not Amyloid Precursor Protein (APP) Intracellular C-Terminal Domain Production Requires a Precisely Oriented APP Dimer Assembled by Transmembrane GXXXG Motifs. J. Biol. Chem. 283, 7733–7744. 10.1074/jbc.m707142200 18201969 PMC2702479

[B28] Kirkitadze M. D. Bitan G. Teplow D. B. (2002). Paradigm Shifts in Alzheimer's Disease and Other Neurodegenerative Disorders: The Emerging Role of Oligomeric Assemblies. J. Neurosci. Res. 69, 567–577. 10.1002/jnr.10328 12210822

[B29] Klein W. Krafft G. A. Finch C. E. (2001). Targeting Small Aβ Oligomers: the Solution to an Alzheimer's Disease Conundrum? Trends Neurosci. 24, 219–224. 10.1016/s0166-2236(00)01749-5 11250006

[B30] Kojro E. Gimpl G. Lammich S. März W. Fahrenholz F. (2001). Low Cholesterol Stimulates the Nonamyloidogenic Pathway by its Effect on the α-secretase ADAM 10. Proc. Natl. Acad. Sci. U.S.A. 98, 5815–5820. 10.1073/pnas.081612998 11309494 PMC33296

[B31] Kumari R. Kumar R. Lynn A. (2014). g_mmpbsa-A GROMACS Tool for High-Throughput MM-PBSA Calculations. J. Chem. Inf. Model. 54, 1951–1962. 10.1021/ci500020m 24850022

[B32] Laura D. Leigh F. Meredith S. C. Straub J. E. Thirumalai D. (2014). Structural Heterogeneity in Transmembrane Amyloid Precursor Protein Homodimer Is a Consequence of Environmental Selection. J. Am. Chem. Soc. 136, 9619–9626. 24926593 10.1021/ja503150x PMC4105063

[B33] Lauritzen I. Pardossi-Piquard R. Bauer C. Brigham E. Abraham J.-D. Ranaldi S. (2012). The -Secretase-Derived C-Terminal Fragment of APP, C99, but Not A , Is a Key Contributor to Early Intraneuronal Lesions in Triple-Transgenic Mouse Hippocampus. J. Neurosci. 32, 16243–16255. 10.1523/jneurosci.2775-12.2012 23152608 PMC5019353

[B34] Li C.-D. Junaid M. Chen H. Ali A. Wei D.-Q. (2019). Helix-switch Enables C99 Dimer Transition between the Multiple Conformations. J. Chem. Inf. Model. 59, 339–350. 10.1021/acs.jcim.8b00559 30570254

[B35] Li C.-D. Xu Q. Gu R.-X. Qu J. Wei D.-Q. (2017). The Dynamic Binding of Cholesterol to the Multiple Sites of C99: as Revealed by Coarse-Grained and All-Atom Simulations. Phys. Chem. Chem. Phys. 19, 3845–3856. 10.1039/c6cp07873g 28102375

[B36] Lorenz C. D. Faraudo J. Travesset A. (2008). Hydrogen Bonding and Binding of Polybasic Residues with Negatively Charged Mixed Lipid Monolayers. Langmuir 24, 1654–1658. 10.1021/la703550t 18211111

[B37] Marrink S. J. de Vries A. H. Tieleman D. P. (2009). Lipids on the Move: Simulations of Membrane Pores, Domains, Stalks and Curves. Biochimica Biophysica Acta (BBA) - Biomembr. 1788, 149–168. 10.1016/j.bbamem.2008.10.006 19013128

[B38] Marrink S. J. Risselada H. J. Yefimov S. Tieleman D. P. De Vries A. H. (2007). The MARTINI Force Field: Coarse Grained Model for Biomolecular Simulations. J. Phys. Chem. B 111, 7812–7824. 10.1021/jp071097f 17569554

[B39] Miyashita N. Straub J. E. Thirumalai D. Sugita Y. (2009). Transmembrane Structures of Amyloid Precursor Protein Dimer Predicted by Replica-Exchange Molecular Dynamics Simulations. J. Am. Chem. Soc. 131, 3438–3439. 10.1021/ja809227c 19275251 PMC2727648

[B40] Monticelli L. Kandasamy S. K. Periole X. Larson R. G. Tieleman D. P. Marrink S.-J. (2008). The MARTINI Coarse-Grained Force Field: Extension to Proteins. J. Chem. Theory Comput. 4, 819–834. 10.1021/ct700324x 26621095

[B41] Munter L.-M. Voigt P. Harmeier A. Kaden D. Gottschalk K. E. Weise C. (2007). GxxxG Motifs within the Amyloid Precursor Protein Transmembrane Sequence Are Critical for the Etiology of Aβ42. EMBO J. 26, 1702–1712. 10.1038/sj.emboj.7601616 17332749 PMC1829382

[B42] Nadezhdin K. D. Bocharova O. V. Bocharov E. V. Arseniev A. S. (2012). Dimeric Structure of Transmembrane Domain of Amyloid Precursor Protein in Micellar Environment. FEBS Lett. 586, 1687–1692. 10.1016/j.febslet.2012.04.062 22584060

[B43] Osenkowski P. Ye W. Wang R. Wolfe M. S. Selkoe D. J. (2008). Direct and Potent Regulation of γ-Secretase by its Lipid Microenvironment. J. Biol. Chem. 283, 22529–22540. 10.1074/jbc.m801925200 18539594 PMC2504869

[B44] Panahi A. Bandara A. Pantelopulos G. A. Dominguez L. Straub J. E. (2016). Specific Binding of Cholesterol to C99 Domain of Amyloid Precursor Protein Depends Critically on Charge State of Protein. J. Phys. Chem. Lett. 7, 3535–3541. 10.1021/acs.jpclett.6b01624 27525349 PMC5293176

[B45] Parrinello M. Rahman A. (1998). Polymorphic Transitions in Single Crystals: A New Molecular Dynamics Method. J. Appl. Phys. 52, 7182–7190.

[B46] Pester O. Barrett P. J. Hornburg D. Hornburg P. Pröbstle R. Widmaier S. (2013). The Backbone Dynamics of the Amyloid Precursor Protein Transmembrane Helix Provides a Rationale for the Sequential Cleavage Mechanism of γ-Secretase. J. Am. Chem. Soc. 135, 1317–1329. 10.1021/ja3112093 23265086 PMC3560327

[B47] Pulina M. Hopkins M. Haroutunian V. Greengard P. Bustos V. (2019). C99, Not Beta-Amyloid, Is Associated with Selective Death of Vulnerable Neurons in Alzheimer's Disease. Alzheimers Dement. 16, 273–282. 10.1016/j.jalz.2019.09.002 31677937

[B48] Refolo L. M. Pappolla M. A. LaFrancois J. Malester B. Schmidt S. D. Thomas-Bryant T. (2001). A Cholesterol-Lowering Drug Reduces β-Amyloid Pathology in a Transgenic Mouse Model of Alzheimer's Disease. Neurobiol. Dis. 8, 890–899. 10.1006/nbdi.2001.0422 11592856

[B49] Runz H. Rietdorf J. Tomic I. de Bernard M. Beyreuther K. Pepperkok R. (2002). Inhibition of Intracellular Cholesterol Transport Alters Presenilin Localization and Amyloid Precursor Protein Processing in Neuronal Cells. J. Neurosci. 22, 1679–1689. 10.1523/jneurosci.22-05-01679.2002 11880497 PMC6758870

[B50] Sapay N. Bennett W. F. D. Tieleman D. P. (2010). Molecular Simulations of Lipid Flip-Flop in the Presence of Model Transmembrane Helices. Biochemistry 49, 7665–7673. 10.1021/bi100878q 20666375

[B51] Sato T. Tang T.-c. Reubins G. Fei J. Z. Fujimoto T. Kienlen-Campard P. (2009). A Helix-To-Coil Transition at the ε-cut Site in the Transmembrane Dimer of the Amyloid Precursor Protein Is Required for Proteolysis. Proc. Natl. Acad. Sci. U.S.A. 106, 1421–1426. 10.1073/pnas.0812261106 19164538 PMC2635791

[B52] Scheuermann S. Hambsch B. Hesse L. Stumm J. Schmidt C. Beher D. (2001). Homodimerization of Amyloid Precursor Protein and its Implication in the Amyloidogenic Pathway of Alzheimer's Disease. J. Biol. Chem. 276, 33923–33929. 10.1074/jbc.m105410200 11438549

[B53] Selkoe D. J. (2019). Early Network Dysfunction in Alzheimer's Disease. Science 365, 540–541. 10.1126/science.aay5188 31395769

[B54] Seubert P. Vigo-Pelfrey C. Esch F. Lee M. Dovey H. Davis D. (1992). Isolation and Quantification of Soluble Alzheimer's β-peptide from Biological Fluids. Nature 359, 325–327. 10.1038/359325a0 1406936

[B55] Shankar G. M. Li S. Mehta T. H. Garcia-Munoz A. Shepardson N. E. Smith I. (2008). Amyloid-β Protein Dimers Isolated Directly from Alzheimer's Brains Impair Synaptic Plasticity and Memory. Nat. Med. 14, 837–842. 10.1038/nm1782 18568035 PMC2772133

[B56] Simons M. Keller P. De Strooper B. Beyreuther K. Dotti C. G. Simons K. (1998). Cholesterol Depletion Inhibits the Generation of β-amyloid in Hippocampal Neurons. Proc. Natl. Acad. Sci. U.S.A. 95, 6460–6464. 10.1073/pnas.95.11.6460 9600988 PMC27798

[B57] Song Y. Hustedt E. J. Brandon S. Sanders C. R. (2013). Competition between Homodimerization and Cholesterol Binding to the C99 Domain of the Amyloid Precursor Protein. Biochemistry 52, 5051–5064. 10.1021/bi400735x 23865807 PMC3758481

[B58] Van Der Spoel D. Lindahl E. Hess B. Groenhof G. Mark A. E. Berendsen H. J. C. (2005). GROMACS: Fast, Flexible, and Free. J. Comput. Chem. 26, 1701–1718. 10.1002/jcc.20291 16211538

[B59] Vashisht K. Verma S. Gupta S. Lynn A. M. Dixit R. Mishra N. (2016). Engineering Nucleotide Specificity of Succinyl-CoA Synthetase in Blastocystis: The Emerging Role of Gatekeeper Residues. Biochemistry 56. 10.1021/acs.biochem.6b00098 PMC5404824 27478903

[B60] Wahrle S. Das P. Nyborg A. C. McLendon C. Shoji M. Kawarabayashi T. (2002). Cholesterol-Dependent γ-Secretase Activity in Buoyant Cholesterol-Rich Membrane Microdomains. Neurobiol. Dis. 9, 11–23. 10.1006/nbdi.2001.0470 11848681

[B61] Walsh D. M. Klyubin I. Fadeeva J. V. Cullen W. K. Anwyl R. Wolfe M. S. (2002). Naturally Secreted Oligomers of Amyloid β Protein Potently Inhibit Hippocampal Long-Term Potentiation *In Vivo* . Nature 416, 535–539. 10.1038/416535a 11932745

[B62] Wang H. Barreyro L. Provasi D. Djemil I. Torres-Arancivia C. Filizola M. (2011). Molecular Determinants and Thermodynamics of the Amyloid Precursor Protein Transmembrane Domain Implicated in Alzheimer's Disease. J. Mol. Biol. 408, 879–895. 10.1016/j.jmb.2011.03.028 21440556 PMC3082318

[B63] Yanez Arteta M. Ainalem M.-L. Porcar L. Martel A. Coker H. Lundberg D. (2014). Interactions of PAMAM Dendrimers with Negatively Charged Model Biomembranes. J. Phys. Chem. B 118, 12892–12906. 10.1021/jp506510s 25310456

[B64] Zott B. Simon M. M. Hong W. Unger F. Chen-Engerer H.-J. Frosch M. P. (2019). A Vicious Cycle of β Amyloid-dependent Neuronal Hyperactivation. Science 365, 559–565. 10.1126/science.aay0198 31395777 PMC6690382

[B65] Zott B. Simon M. M. Hong W. Unger F. Chen-Engerer H.-J. Frosch M. P. (2019). A Vicious Cycle of β Amyloid-dependent Neuronal Hyperactivation. Science 365, 559–565. 10.1126/science.aay0198 31395777 PMC6690382

