# Supplementary material for: Effect of Cholesterol on C99 Dimerization: Revealed by Molecular Dynamics Simulations
Source: Front Mol Biosci. 2022 Jul 19;9:872385. doi: 10.3389/fmolb.2022.872385 (PMC9343951; doi:10.3389/fmolb.2022.872385)
Supplement: Supplementary file 1 [file DataSheet1.doc]

**Supporting Information**


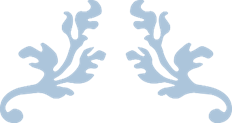


**Effect of Cholesterol on C99 Dimerization: Revealed by MD Simulations**

*Cheng-Dong Li^1^, Muhammad Junaid^1^, Xiaoqi Shan^1^, Yanjing Wang^1^, Xiangeng Wang^1^, Abbas Khan^1^, Dong-Qing Wei^1,2^**

1. State Key Laboratory of Microbial Metabolism, Joint Laboratory of International Cooperation in Metabolic and Developmental Sciences, Ministry of Education, Department of Bioinformatics and Biological Statistics, School of Life Sciences and Biotechnology, Shanghai Jiao Tong University, Shanghai, 200240, P.R. China.
2. Peng Cheng Laboratory, Vanke Cloud City Phase I Building 8, Xili Street, Nanshan District, Shenzhen, Guangdong, 518055, China

E-mail address: [dqwei@sjtu.edu.cn](mailto:dqwei@sjtu.edu.cn)


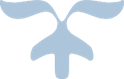


**Table S1.** Summary of simulations

| **Membrane Compositions** | **Cholesterol concentration** | **Protein structure** | **Simulation types** | **Number of samples** |
| --- | --- | --- | --- | --- |
| POPC: POPG=3:1 | 0 % | Two Monomers | 3 μs CG | 20 |
| POPC: POPG=3:1 | 20 % | Two Monomers | 3 μs CG | 20 |
| DPPC | 0 % | Two Monomers | 3 μs CG | 20 |
| DPPC | 20 % | Two Monomers | 3 μs CG | 20 |
| DPPC | 0 % | GW2 | 3 μs CG | 3 |
| DPPC | 20 % | GW2 | 3 μs CG | 3 |
| DPPC | 0 % | GN2 | 3 μs CG | 3 |
| DPPC | 20 % | GN2 | 3 μs CG | 3 |
| DPPC | 0 % | GIN | 3 μs CG | 3 |
| DPPC | 20 % | GIN | 3 μs CG | 3 |
| DPPC | 0 % | GOUT | 3 μs CG | 3 |
| DPPC | 20 % | GOUT | 3 μs CG | 3 |
| DPPC | 0 % | GSIDE1 | 3 μs CG | 3 |
| DPPC | 20 % | GSIDE1 | 3 μs CG | 3 |
| DPPC | 0 % | GD1 | 3 μs CG | 3 |
| DPPC | 20 % | GD1 | 3 μs CG | 3 |
| DPPC | 20 % | GW2 | 3 μs restraint CG | 1 |
| DPPC | 20 % | GOUT | 3 μs restraint CG | 1 |
| DPPC | 20 % | GIN | 3 μs restraint CG | 1 |
| DPPC | 20 % | GSIDE1 | 3 μs restraint CG | 1 |
| DPPC | 20 % | GN2 | 3 μs restraint CG | 1 |
| DPPC | 20 % | GD1 | 3 μs restraint CG | 1 |
| DPPC | 0 % | GW2 | 100 ns AT | 1 |
| DPPC | 20 % | GW2 | 100 ns AT | 1 |
| DPPC | 20 % | Two Monomers | 3 μs AT | 1 |

**Table S2.** The MM Energy of GW2 with and without cholestrol.

| **C99 dimer**  **(GW2)** | **Van der Waal (kJ/mol)** | **Electrostatic (kJ/mol)** | **MM (kJ/mol)** |
| --- | --- | --- | --- |
| **GW2** | **-579.655±78.741** | **393.813±83.181** | **-185.842±100.007** |
| **Without cholesterol** | **-249.392±21.919** | **118.884±167.117** | **-130.508±185.200** |
| **With 20% cholesterol** | **-273.500±37.409** | **-296.001±128.095** | **-569.501±107.697** |

∆E_MM_ is the sum of van der Waals and electrostatic interactions, averaged by 20 calculations by g_mmpbsa with an interval of 2ns from 100ns AT simulations.


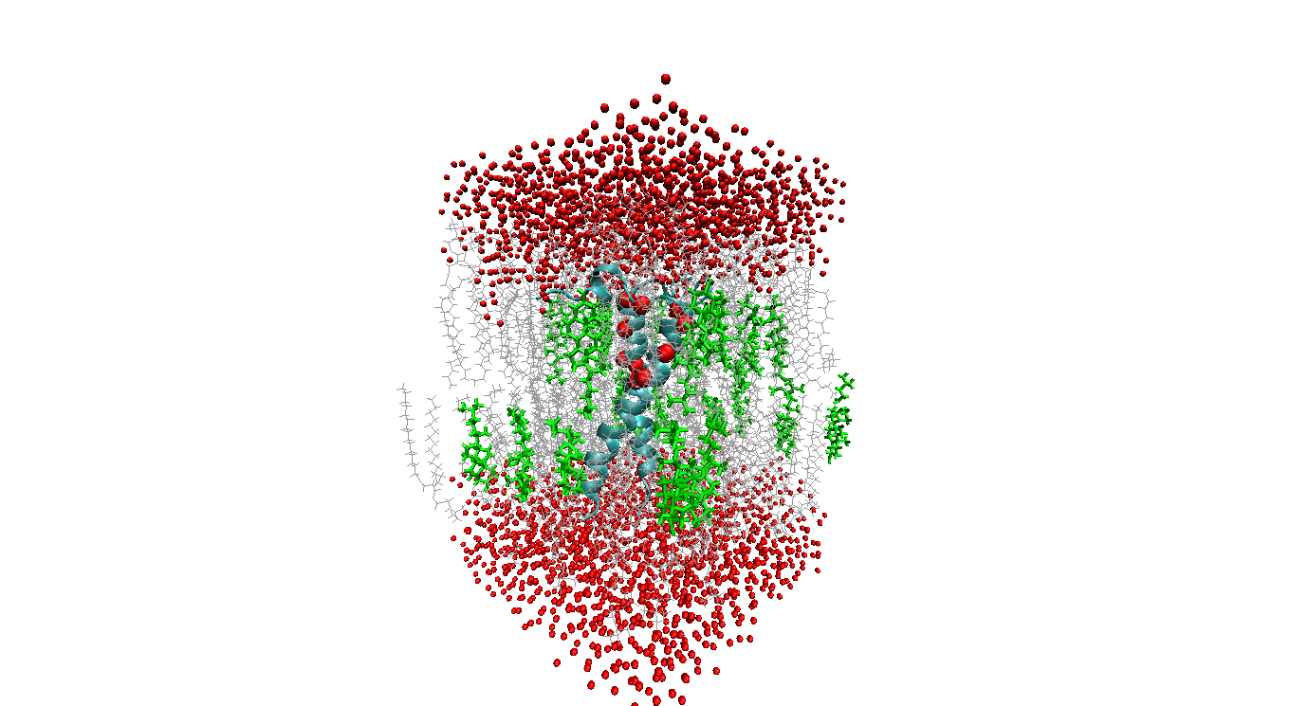

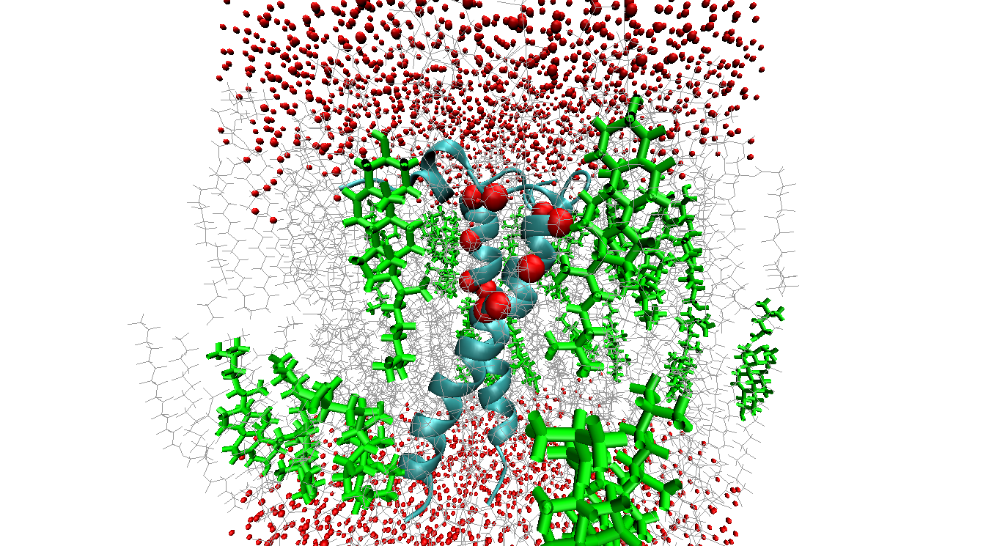


Figure S1. Two separated C99 monomers form a dimer in DPPC bilayer containing 20% cholesterol at 310K, which was observed in the 3μs all-atom (AT) simulation. The initial centroid distance of the two C99 monomers was set to ~3 nm. The right image is a partial enlargement. Protein is represented by cartoon model; cholesterol molecules are shown in green and lipid molecules are shown in gray.


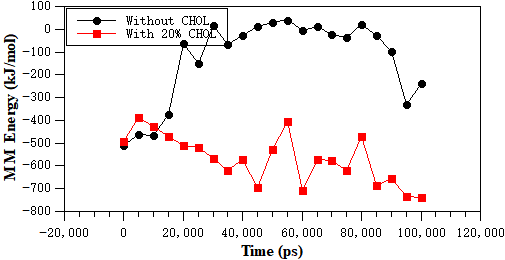


Figure S2. The MM Energy of GW2 with and without CHOL. ∆E_MM_ energy is the sum of van der Waals and electrostatic interactions, which is averaged from 20 calculations by g_mmpbsa with an interval of 2ns in 100ns AT simulations, starting from the same GW2 conformation.


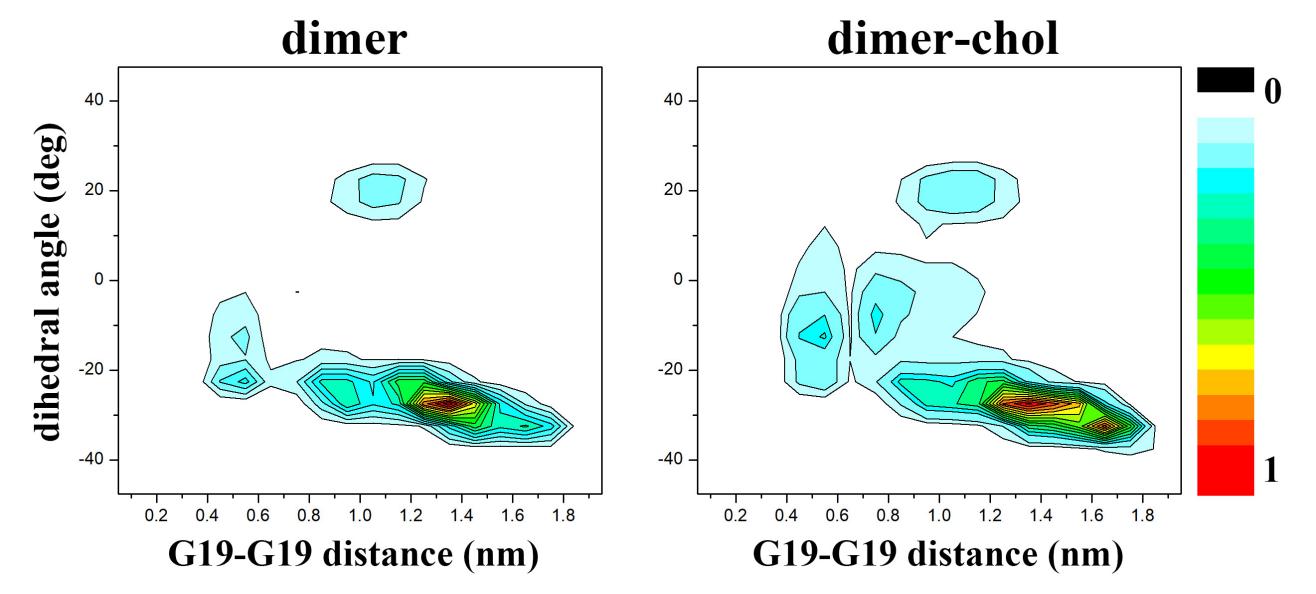
Figure S3. The merged distributions of figure 4. The left is the merged distribution of C99 dimer when without cholesterol, the right is the merged distribution of C9 dimer when with 20% cholesterol.

Interestingly, the total conformation distributions from the simulations starting with six representative conformations (Figure S3), are consistent to the conformation distributions from the simulations starting with two initial spatially separated monomers (Figure 6), no matter with or without cholesterol. This proved that our results are robust. The finite time simulation sampling is highly dependent on the initial structure; therefore, it is a better system setting to employ two spatially separated monomers as the initial conformation in the simulation of protein dimerization.

Abbreviations:

DPPC: dipalmitoyl-phosphatidylcholine

POPC: 1-palmitoyl-2-oleoyl-phosphatidylcholine

POPG: 1-palmitoyl-2-oleoyl-phosphatidylglycerol
